# RCAN1 in cardiovascular diseases: molecular mechanisms and a potential therapeutic target

**DOI:** 10.1186/s10020-020-00249-0

**Published:** 2020-12-02

**Authors:** Shuai Wang, Yuqing Wang, Kaixin Qiu, Jin Zhu, Yili Wu

**Affiliations:** 1 Shandong Collaborative Innovation Center for Diagnosis, Treatment and Behavioral Interventions of Mental Disorders, Institute of Mental Health, Jining Medical University, Jianshe South Road No. 45, Rencheng District, Jining, 272013 Shandong China; 2 Shandong Key Laboratory of Behavioral Medicine, School of Mental Health, Jining Medical University, Jianshe South Road No. 45, Rencheng District, Jining, 272013 Shandong China; 3 Cheeloo College of Medicine, Shandong University, Wenhua West Road No. 44, Lixia District, JinanShandong, 250012 China

**Keywords:** Cardiovascular disease, Regulator of calcineurin 1, Therapeutic potential

## Abstract

Cardiovascular diseases (CVDs) are the leading cause of mortality worldwide. Considerable efforts are needed to elucidate the underlying mechanisms for the prevention and treatment of CVDs. Regulator of calcineurin 1 (RCAN1) is involved in both development/maintenance of the cardiovascular system and the pathogenesis of CVDs. RCAN1 reduction protects against atherosclerosis by reducing the uptake of oxidized low-density lipoproteins, whereas RCAN1 has a protective effect on myocardial ischemia/reperfusion injury, myocardial hypertrophy and intramural hematoma/aortic rupture mainly mediated by maintaining mitochondrial function and inhibiting calcineurin and Rho kinase activity, respectively. In this review, the regulation and the function of RCAN1 are summarized. Moreover, the dysregulation of RCAN1 in CVDs is reviewed. In addition, the beneficial role of RCAN1 reduction in atherosclerosis and the protective role of RCAN1 in myocardial ischemia/reperfusion injury, myocardial hypertrophy and intramural hematoma /aortic rupture are discussed, as well as underlying mechanisms. Furthermore, the therapeutic potential and challenges of targeting RCAN1 for CVDs treatment are also discussed.

## Background

Cardiovascular diseases (CVDs), including heart and vascular disorders, are major causes of mortality and disability globally, especially in developed countries. CVDs result in estimated 17.7 million deaths annually and account for 20% and 24% of total age-standardized global burden of disease in women and men respectively (Thomas et al. 2018). In 2013, the World Health Organization launched the Global Action Plan which focused on health services support and public policy, aiming to prevent and manage four non-communicable diseases and CVDs are the major one (Organization 2013). Elucidating the molecular mechanisms of the pathogenesis of CVDs is essential for their prevention and treatment. Accumulated evidence indicates that regulator of calcineurin 1 (RCAN1) plays pivotal roles in the pathogenesis of CVDs and may be a potential therapeutic target.

RCAN1 functions in multiple physiological processes, including the development and functional maintenance of the cardiovascular system. For example, genetic evidence revealed that several *RCAN1* mutations increased the risk of congenital heart disease (Guo et al. 2015; Li et al. 2018, 2015). Mounting evidence indicates that RCAN1 exerts different effect in various CVDs. RCAN1 reduction is beneficial to atherosclerosis (Mendez-Barbero et al. 2013) whereas RCAN1 protects against myocardial ischemia/reperfusion injury (Rotter et al. 2014), myocardial hypertrophy (Sussman et al. 1998) and intramural hematoma /aortic rupture (Villahoz et al. 2018). Moreover, growing evidence indicates that RCAN1 is dysregulated in CVDs. For example, remarkable increase of RCAN1 was observed in atherosclerosis lesions in patients and mice (Mendez-Barbero et al. 2013). Therefore, we summarize the current knowledge of the regulation and function of RCAN1, and discuss the dysregulation of RCAN1 in CVDs and its role in the pathogenesis of CVDs, aiming to propose the therapeutic potential of targeting RCAN1 for the treatment of CVDs. Importantly, the critical issues for drug development by targeting RCAN1 are also discussed.

## Overview of RCAN1

**Fig. 1:**
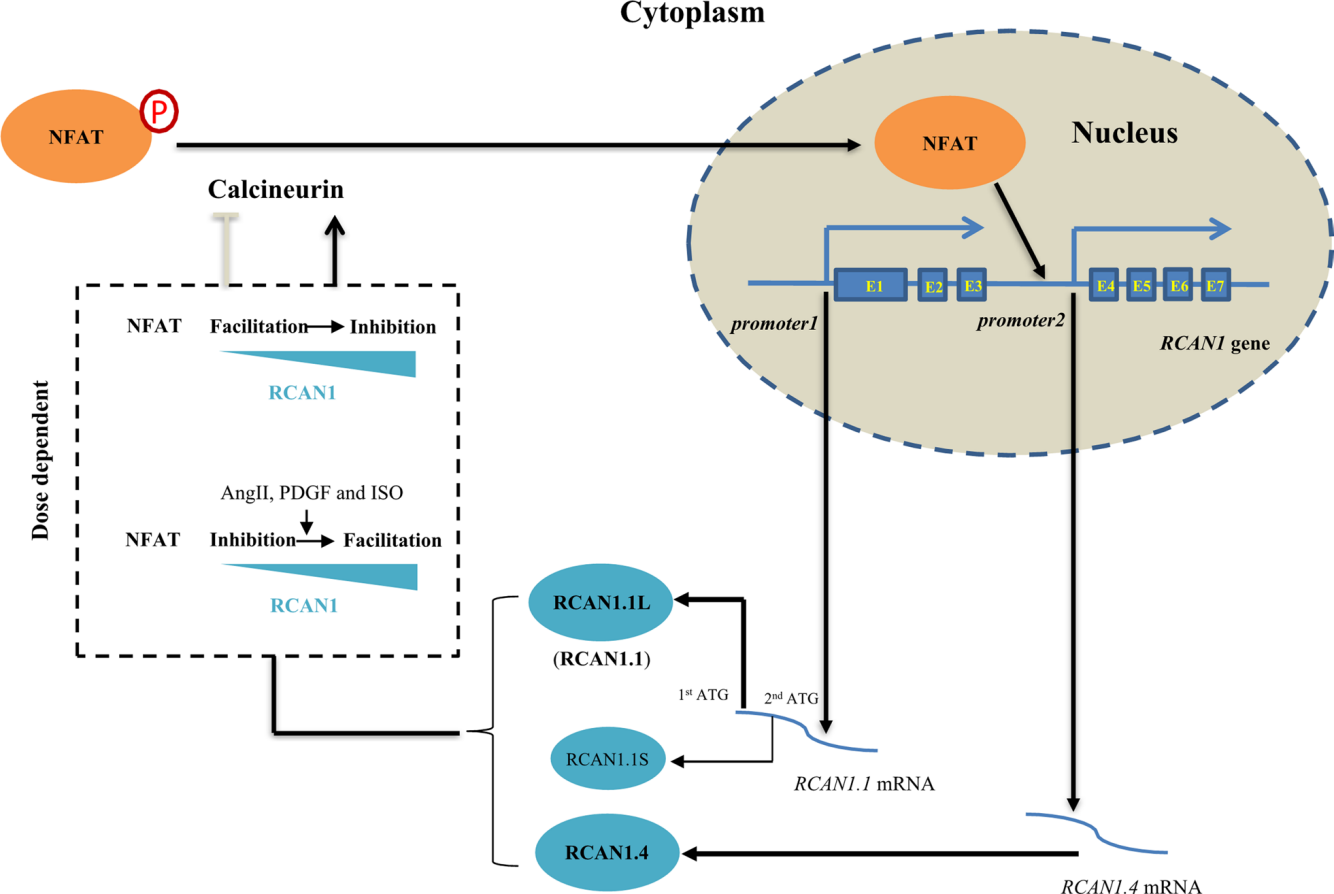
The bidirectional role of RCAN1 in calcineurin/NFAT signaling pathway. The *RCAN1* gene consists of seven exons (presented as E1–E7). *RCAN1.1* (E1, E5–E7) and *RCAN1.4* (E4, E5–E7) are the two major transcripts, which are generated by alternative promoter using and splicing. Two isoforms, RCAN1.1L (252 amino acids) and RCAN1.1S (197 amino acids), are generated from *RCAN1.1* by alternative usage of two in-frame translational start codons (presented as 1st ATG and 2nd ATG). As RCAN1.1L is the dominant isoform of RCAN1.1, hence RCAN1.1 hereafter stands for RCAN1.1L. RCAN1.4, another relative abundant isoform is translated from *RCAN1.4*. Each isoform participates in calcineurin-NFAT signaling by regulating calcineurin-mediated NFAT dephosphorylation, while active NFAT facilitates *RCAN1.4* transcription. RCAN1 plays complex roles in the regulation calcineurin-NFAT signaling in a dose-dependent manner. Low level of RCAN1 is essential for the maintenance of calcineurin-NFAT signaling while high dose of RCAN1 exerts inhibitory effect on calcineurin-NFAT signaling. However, in response to AngII, PDGF or ISO treatment, RCAN1 functions as an inhibitor of calcineurin-NFAT signaling when its level is low, but as a facilitator of calcineurin-NFAT signaling when its level is high. The arrowhead stands for positive regulation, while T-shaped end stands for inhibition. P within the red circle represents the phosphorylation form of NFAT (inactive form)

The *RCAN1* gene is located in the q22.1–q22.2 region of human chromosome 21 and is expressed primarily in the brain, heart, skeletal muscle and endocrine tissues including the adrenal gland and pancreas (Ermak et al. 2001; Fuentes et al. 1997, 1995; Peiris et al. 2012). The *RCAN1* gene consists of seven exons separated by six introns. Four transcripts are generated by alternative promoter using and splicing, among which *RCAN1.1* (exon1, exon5, exon6, exon7) and *RCAN1.4* (exon4, exon5, exon6, exon7) are the two major transcripts (Wu et al. 2014) (Fig. 1). *RCAN1.1* is highly expressed in the brain, heart and skeletal muscle. Two isoforms, RCAN1.1L (252 amino acids) and RCAN1.1S (197 amino acids), are generated from transcript *RCAN1.1* by alternative usage of two in-frame translational start codons (Fig. 1). RCAN1.1L is the major isoform, while the level of RCAN1.1S is extremely low (Wu and Song 2013). Thus, RCAN1.1L is hereafter referred as RCAN1.1. RCAN1.4, translated from transcript *RCAN1.4*, is mainly expressed in the heart and skeletal muscle. All the isoforms share the same sequence of 168 amino acids at C-terminus, in which multiple post-translational modification sites are located.

The expression of RCAN1 is regulated at both transcriptional and post-translational levels. First, *RCAN1.1* is up-regulated by glucocorticoids at transcriptional level as the promoter region of *RCAN1.1* contains a functional glucocorticoid response element (GRE) (Sun et al. 2011; Shen et al. 2004; Yoshida et al. 2002). VEGF increases *RCAN1.1* transcription via the binding of TEF3 isoform 1 to M-CAT sites in *RCAN1.1* promoter (Liu et al. 2011, 2008; Qin et al. 2006). The expression of RCAN1.4 is sensitive to NFAT signaling as 15 potential NFAT binding sites are within the promoter region of *RCAN1.4* (Sun et al. 2014b; Yang et al. 2000). In addition, NFATs act in cooperation with other transcription factors to achieve synergistic activation of *RCAN1.4* transcription, e.g. CCAAT/enhancer binding protein (C/EBPβ), AP-1, ATF6, NF-κB etc., which have been reviewed previously (Wu et al. 2014). Moreover, RCAN1 is degraded by both ubiquitin–proteasome pathway and chaperone-mediated autophagy lysosome pathway, which is regulated by the post-translational modification, including phosphorylation, ubiquitination, acetylation and neddylation (Han et al. 2014; Noh et al. 2012).

One of the main molecular functions of RCAN1 is generally known to regulate calcineurin-NFAT signaling which functions in CVDs. NFAT proteins are primarily located in the cytoplasm until the calcium-dependent calcineurin activation stimulates their dephosphorylation. The dephosphorylated NFATs further activate the transcription of target genes, including *RCAN1.4*. As an important endogenous regulator of calcineurin, RCAN1 has bidirectional roles in the regulation of calcineurin-NFAT signaling (Shin et al. 2011; Wu et al. 2014). Increased RCAN1 inhibits calcineurin-NFAT signaling in endothelial cells and myocardium in vitro and in vivo, whereas RCAN1 knockout also leads to the impairment of calcineurin-NFAT signaling (Fig. 1) (Sanna et al. 2006; Vega et al. 2003). It indicates that low level of RCAN1 is essential for the maintenance of calcineurin-NFAT signaling in cardiac system, while increased RCAN1 inhibits NFAT signaling. However, after angiotensin II (AngII), platelet-derived growth factor (PDGF) or isoproterenol (ISO) treatment, RCAN1 functions as an inhibitor of calcineurin-NFAT signaling in a dose-dependent manner when its level is low, while it functions as a facilitator when its level is high (Fig. 1)(Shin et al. 2011). It indicates that RCAN1 plays a complex role in regulating calcineurin-NFAT signaling, depending on both the dosage of RCAN1 and the environmental levels of AngII, PDGF and ISO.

**Fig. 2:**
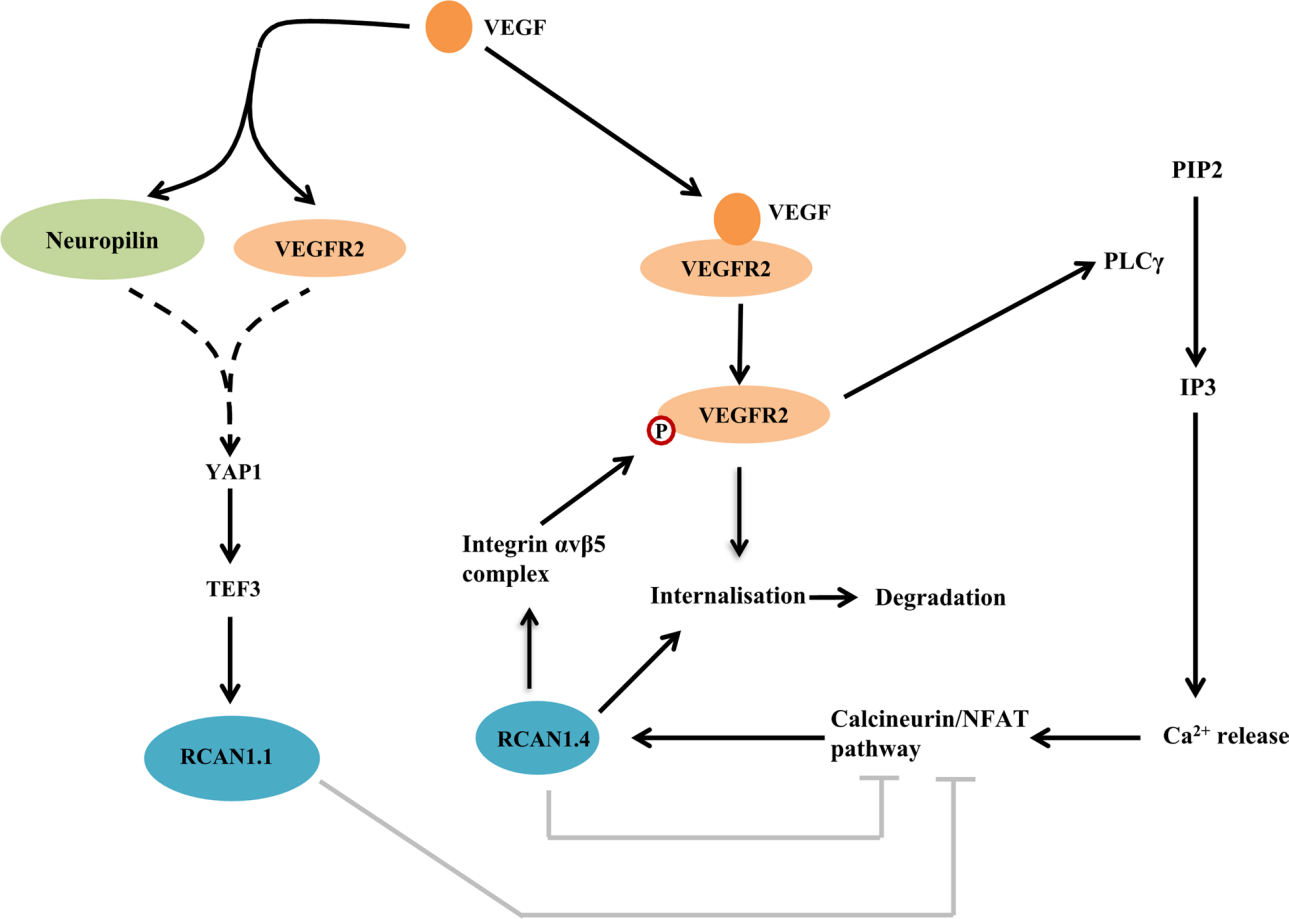
RCAN1 in the regulatory network of VEGF-VEGFR2 signaling. VEGF induces RCAN1.1 upregulation via YAP1-TEF3 activation which is possibly mediated by neuropilin and VEGFR2. VEGF stimulation-induced VEGFR2 phosphorylation activates PLCγ, which cleavages PIP2 into IP3. IP3 enhances the release of Ca^2+^ contributing to the activation of calcineurin/NFAT signaling, which promotes RCAN1.4 expression. On the other hand, both RCAN1.1 and RCAN1.4 participates in VEGF-VEGFR2 signaling via inhibiting calcineurin-NFAT signaling-mediated RCAN1.4 expression. RCAN1.4 facilitates VEGFR2 internalisation followed by proteolytic degradation, and facilitatesVEGFR2 phosphorylation possibly mediated by integrin αvβ5 complex. The arrowhead stands for positive regulation, while T-shaped end stands for inhibition. Dotted line stands for possible signaling pathways. P within the red circle represents the phosphorylation form of VEGFR2

VEGF-VEGFR2 signaling plays crucial roles in the cardiovascular system (Holmes et al. 2007). RCAN1 is implicated in the regulation of VEGF-VEGFR2 signaling via various mechanisms (Fig. 2). First, RCAN1.4 facilitates VEGF-stimulated VEGFR2 internalization and VEGFR2 dependent endothelial cell migration and angiogenesis (Alghanem et al. 2017). In addition, RCAN1.4 also participates in endothelial cell migration through interaction with integrin αvβ3 complex which facilitates VEGFR2 phosphorylation (Iizuka et al. 2004). Moreover, RCAN1.1 functions downstream of VEGF promoting endothelial cell proliferation and angiogenesis through the activation of calcineurin-NFAT pathway (Qin et al. 2006) (Fig. 2). Furthermore, VEGF regulates the transcription of *RCAN1.1* and *RCAN1.4* via different pathways, forming a feedback loop with VEGF signaling (Fig. 2). For example, in vascular endothelial cells, VEGF facilitates the up-regulation of *RCAN1.1* by promoting the binding of TEF3 to the M-CAT cis-element in the *RCAN1.1* promoter, while TEF3 activation is facilitated by its interaction with Yes1 associated transcriptional regulator (YAP1)(Cui et al. 2020; Liu et al. 2011; Qin et al. 2006). YAP1 is commonly known to be activated by VEGF via two receptors, neuropilin and VEGFR2 (Fig. 2) (Elaimy and Mercurio 2018). Besides, RCAN1.4 is also up-regulated by VEGF-VEGFR2 signaling. The binding of VEGF to VEGFR2 results in VEGFR2 autophosphorylation at the tyrosine 1175 residue (Uniewicz et al. 2011). The phosphorylated VEGFR2 activates phospholipase C-γ(PLCγ), which catalyzes phosphatidylinositol (4,5)-bisphosphate (PIP2) into inositol (1,4,5)-trisphosphate (IP3) and diacylglycerol (DAG) (Takahashi et al. 2001). IP3 stimulates Ca^2+^ release from the endoplasmic reticulum (ER), subsequently activating calcineurin-NFAT-mediated *RCAN1.4* transcription (Fig. 2).

## RCAN1 in cardiovascular disorders

### Atherosclerosis

Atherosclerosis, the leading cause of CVDs, is characterized by the development of atheromatous plaques (Lusis 2000). Atheromatous plaque development begins with the subendothelial accumulation of apolipoprotein B-containing low-density lipoproteins (LDLs). Oxidized LDL (oxLDL) triggers the activation of the vascular endothelium and drives differentiation of monocytes into macrophages in vascular intima (Hansson and Hermansson 2011). After engulfing oxLDL, macrophages shift to a more sessile foam-cell phenotype in an atherosclerotic plaque. Along with the plaque expansion and the lesion maturation, the vascular wall is remodeled. Thus, the wall of the blood vessel is thickened, the lumen of the arteries is narrowed and the elasticity of the artery walls is lost. The terminal manifestations of atherosclerosis, atherosclerotic plaque rupture, vascular stenosis and atherothrombosis, could result in acute cardiovascular events, while atherothrombosis is the most common cause of acute cardiovascular events (Baron et al. 2016; Viles-Gonzalez et al. 2004).

Remarkable increase of RCAN1.4 was observed in human and mouse atherosclerotic arteries. Although RCAN1.1 appeared to be higher in atherosclerotic lesions, the difference was less marked compared with RCAN1.4, especially in mice models. Atherogenic oxLDL was demonstrated to induce RCAN1.4 but not RCAN1.1 expression in macrophages, endothelial cells and vascular smooth muscle cells. In contrast to RCAN1.4 upregulation, a slight increase in RCAN1.1 expression in atherosclerotic plaques might be attributed to the recruitment of other cell populations with constitutively high RCAN1.1 expression (Mendez-Barbero et al. 2013).

Altered expression of RCAN1 is implicated in the pathogenesis and progress of atherosclerosis. Genetic deletion of *Rcan1* significantly reduced the extent and severity of atherosclerosis in ApoE knockout mice with a high-fat diet. CD36 is an oxLDL receptor which functions in oxLDL uptake and foam-cell formation (Endemann et al. 1993; Nakata et al. 1999). *Rcan1* deletion can significantly decrease CD36 level in the aortic arches while complementary expression of both Rcan1.1 and Rcan1.4 could concomitantly increase the numbers of oxLDL particles taken up by macrophages, indicating the protective effect of RCAN1 downregulation on atherosclerosis might be mediated by CD36 reduction (Mendez-Barbero et al. 2013). Additionally, VEGF signaling promotes the endothelial cell proliferation (Carmeliet et al. 1999), macrophage infiltration (Guo et al. 2018) and foam cell formation (Yan et al. 2017), which all play pivotal roles in the pathogenesis of atherosclerosis. As a crucial modulator of VEGFR2, the reduction of RCAN1.1 and RCAN1.4 might be protective for atherosclerosis by inhibiting VEGFR2 mediated VEGF signaling (Fig. 2) (Alghanem et al. 2017). Above evidence suggested that RCAN1 inhibition may have therapeutic potential for slowing the progression of atherosclerosis by inhibiting VEGF-VEGFR2 signaling.

On the other hand, it has been proposed that increased RCAN1 may have protective effect on atherosclerosis based on the fact that patients with Down syndrome (trisomy of chromosome 21) have a low incidence of atherosclerosis and hypertension, a risk factor for atherosclerosis, and the *RCAN1* gene is located on chromosome 21 (de Asua et al. 2015; Murdoch et al. 1977; Rodrigues et al. 2011; Roy-Vallejo et al. 2020; Ylä-Herttuala et al. 1989). It has to be noted that more than a hundred genes are located on chromosome 21, which may contribute to the protective effect on atherosclerosis and hypertension, not only the RCAN1 gene. It has never been demonstrated that increased RCAN1 has protective effect on atherosclerosis and hypertension, whereas increased expression of the superoxide dismutase (SOD) gene, another gene on chromosome 21, has been demonstrated to contribute to the protective effect on atherosclerosis (t Hoen et al. 2003; Yang et al. 2009). Moreover, genetic deletion of *Rcan1* significantly reduced the extent and severity of atherosclerosis in mice, which directly demonstrated that RCAN1 reduction has beneficial effect on atherosclerosis (Mendez-Barbero et al. 2013). In addition, RCAN1 has bidirectional roles in the regulation of calcineurin-NFAT signaling (Shin et al. 2011; Wu et al. 2014). Increased RCAN1 inhibits calcineurin-NFAT signaling, whereas RCAN1 knockout also leads to the impairment of calcineurin-NFAT signaling (Fig. 1) (Sanna et al. 2006; Vega et al. 2003). Although NFAT-mediated vascular contractility and stiffness were found to be increased in *Rcan1* knockout mice, it remains unclear whether RCAN1 could reduce vascular contractility and stiffness (Garcia-Redondo et al. 2018). Importantly, no direct evidence shows that RCAN1 plays a beneficial role in hypertension and atherosclerosis. Current evidence indicates that RCAN1 reduction is beneficial to atherosclerosis, which may have therapeutic potential for slowing or delaying the progression of atherosclerosis by inhibiting CD36 and VEGF signaling.

### Myocardial infarction

Acute myocardial infarction (AMI) is a type of acute coronary syndrome in which sudden blockage of a coronary artery results in myocardial ischemic injury (Hashmi and Al-Salam 2015). Atherosclerotic plaque disruption with subsequent formation of thromboembolism is the major cause of myocardial infarction (Baron et al. 2016; Viles-Gonzalez et al. 2004). The most effective therapeutic intervention is timely myocardial reperfusion by using thrombolytic therapy or primary percutaneous coronary intervention to salvage the ischemic myocardium. Although the reperfusion is critical for myocardial survival, it does cause ‘reperfusion injury’.

Enhanced NFAT activity and subsequently induced expression of Rcan1.4 rather than Rcan1.1 were confirmed in the infarct area (Echtermeyer et al. 2011; Rotter et al. 2014). The levels of *Rcan1.4* transcript and protein fluctuate with a circadian rhythm and the peak expression of Rcan1.4 is associated with the greatest resistance to myocardial ischemia/reperfusion (I/R) injury (Rotter et al. 2014). Consistently, cardiac-specific overexpression of RCAN1.4 protects against cardiac I/R, whereas *Rcan1* deletion exacerbates I/R injury (Parra et al. 2018; Rotter et al. 2014; van Rooij et al. 2004). It suggested that RCAN1 might play a protective role in myocardial I/R injury.

The protective effect of RCAN1 on I/R injury is partially mediated by inhibiting calcineurin activity, which is further supported by that FK506, a calcineurin inhibitor, confers protection to myocardial infarction in *Rcan1* KO mice (Rotter et al. 2014). Moreover, RCAN1 protects heart from I/R damage by modulating mitochondrial function. RCAN1 deletion impairs mitochondrial function and exacerbates myocardial I/R injury (Rotter et al. 2014). Mitochondrial fission/fusion and autophagy (termed as mitophagy) are the two critical determinants for mitochondrial function. Mitochondrial fusion and fission work in concert to maintain the shape, size, and number of mitochondria and their physiological function. Mitochondrial fragmentation caused by fission was a noteworthy feature of I/R injury, while inhibiting mitochondrial fission has a protective effect on I/R injury (Ong et al. 2010). RCAN1.1 is demonstrated to impede mitochondrial fission and maintain a more fused mitochondrial network in cardiomyocytes, suggesting that the protective effect of RCAN1.1 on myocardial I/R injury may be mediated by suppressing mitochondrial fission (Corbalan and Kitsis 2018; Parra et al. 2018). Mitochondrial fission is initiated by GTPase dynamin-related protein 1 (DRP1), while the subsequent activation of mitofusin 1 (Mfn1), mitofusin 2 (Mfn2) and optic atrophy 1(OPA1), three large GTPases, reversely suppresses mitochondrial fission. RCAN1 inhibits mitochondrial fission by attenuating calcineurin-dependent DRP1 dephosphorylation and maintaining OPA1 and Mfn2 levels (Cereghetti et al. 2008; Corbalan and Kitsis 2018). On the other hand, RCAN1.1 is beneficial for the maintenance of mitochondrial function by accelerating mitophagy in response to myocardial I/R damage (Duan et al. 2015; Sun et al. 2014a; Yan et al. 2014). Under hypoxic conditions, mitochondria in cardiomyocytes are extremely vulnerable, while dysfunctional mitochondria is detrimental for cells due to imbalanced release of reactive oxygen species (ROS), activation of apoptotic pathway and cell growth inhibition (Scherz-Shouval and Elazar 2011; Sun et al. 2014a; Yan et al. 2014). Therefore, efficient removal of dysfunctional mitochondria by mitophagy is critical for cell protection under hypoxic stimuli. Parkin, a E3 ubiquitin ligase, is a specific mitophagy receptor, which is recruited to the mitochondria by PINK1 (PTEN-induced kinase protein 1) to ubiquitylate proteins involved in mitophagy. RCAN1.1 promotes mitophagy by increasing PINK1 expression and facilitating PINK1-mediated translocation of Parkin into mitochondria. Thus, the effect of RCAN1.1 on mitochondrial fission and mitophagy plays a beneficial role in protecting heart from I/R injury, while the role of RCAN1.4 in mitophagy needs to be investigated. It highly suggests that modulating RCAN1-mediated mitochondrial function in cardiomyocytes has a therapeutic potential for the treatment of myocardial I/R injury.

### Aortic intramural hematoma

Intramural hematoma (IMH) is a life-threatening acute aortic disease. Pathological vascular wall remodeling involving structural and functional alterations is a central feature of IMH. IMH is a contained hematoma featuring bleeding within the medial layer, which weakens the aortic wall homeostasis, therefore, long-duration IMH always progresses to aortic aneurysm or pseudoaneurysm and aortic dissection or rupture (Evangelista et al. 2003).

Growing evidence suggests that RCAN1 is implicated in the pathogenesis of IMH. First, AngII induces IMH and contributes to aneurysm formation in the ascending and the abdominal aorta in animal models (Rateri et al. 2014), while NFAT activation and increased RCAN1.4 expression are induced by AngII in the aorta (Esteban et al. 2011). Moreover, the conditional deletion of *Rcan1* in smooth muscle cells (SMCs) or endothelial cells (ECs) disrupts aortic wall homeostasis, predisposing the aorta to hypertension-induced IMH, and subsequent aneurysm and rupture (Villahoz et al. 2018). Rho kinase (ROCK)-mediated myosin light chain (MLC) phosphorylation plays important roles in various cellular processes such as cellular morphogenesis, motility, and smooth muscle contraction, thus, ROCK hyper-activation-induced sustained MLC phosphorylation may contribute to the pathogenesis of vascular remodeling-related diseases(Kaneko-Kawano et al. 2012). RCAN1 is proved to inhibit the activity of GSK3β. The loss of GSK3β inhibition in conditional *RCAN1*^−/−^ cells promotes ROCK activation and subsequently increases MLC phosphorylation, which further alters aortic wall structure and predisposes to IMH and aortic rupture (Villahoz et al. 2018). These results indicate that the maintenance of RCAN1 level and activity is beneficial for the prevention or treatment of IMH.

### Cardiac hypertrophy

Cardiac growth is an adaptive physiological process that satisfies the hemodynamic needs by modulating cardiac output. Under pathological conditions, e.g., chronic hypertension and aortic stenosis, aberrant cardiac growth with the consequence of imbalanced myocardium contractility and consumption leads to heart decompensation and failure. Thus, pathological hypertrophy caused by eccentric cardiac remodeling differs from physiological hypertrophy as only pathological hypertrophy leads to heart failure (Burchfield et al. 2013; Hunter and Chien 1999; Xie et al. 2013).

The level of *Rcan1.1* transcript is increased by 2.97-fold in pathological hypertrophy model mice compared with that in physiological hypertrophy model mice (Song et al. 2012). Moreover, weighted gene co-expression network analysis (WGCNA) revealed that the *RCAN1* gene is a top hub gene involved in cardiac hypertrophy (Tan et al. 2013). Calcineurin activation is a key mediator of stress-induced cardiac hypertrophy and consistently, calcineurin suppression is beneficial for pathological hypertrophy (Molkentin et al. 1998; Zou et al. 2001). For example, isoproterenol-induced myocardial hypertrophy was inhibited by four-and-a-half LIM domain family protein 2 (FHL2) via suppressing calcineurin activation (Hojayev et al. 2012). Calcium/calmodulin dependent protein kinase II (CaMKII) phosphorylates CnA at Ser411, leading to the inhibition of calcineurin activity and anti-hypertrophic effect (Kreusser et al. 2014). The transgenic expression of calcineurin inhibitory domains of Cain/Cabin-1 and A-kinase anchoring protein 79 inhibits cardiac hypertrophy in response to catecholamine infusion or pressure overload (De Windt et al. 2001). Calcineurin inhibitors, cyclosporine A (CyA) and FK506, also prevented cardiac hypertrophy via blunting NFAT signaling in a mouse model (Sussman et al. 1998). Therefore, as an endogenous inhibitor of calcineurin, RCAN1 may act against cardiac hypertrophy by regulating calcineurin-dependent pathways.

### Cardiac valve dysplasia

The cardiac valves include aortic and pulmonic semilunar valves at the arterial pole as well as mitral and tricuspid valves separating the atria and ventricles. Cardiac valve dysplasia is a major type of congenital heart disorders and a major cause of valvular heart disease, including mitral or tricuspid valve insufficiency, pulmonary or aortic valve stenosis etc. The clinical manifestation includes fatigue, chest congestion, chest pain, shortness of breath, vertigo, syncope or symptoms of heart failure depending on the severity of valve dysplasia (Hoffman and Kaplan 2002; Pierpont et al. 2007).

The process of valve development is conserved, which can be explained by epithelial-to-mesenchymal transition of endocardium. It consists of three steps, the formation of endocardial cushions, the growth of endocardial cushions and valve primordial, and valve maturation by extracellular matrix deposition and remodeling (Combs and Yutzey 2009). RCAN1.4 is highly expressed in endocardial cushions during heart development (Wu et al. 2007), while *RCAN1.4* silencing increases the migration of valve endothelial cells, a key process of valvuogenesis (Jang et al. 2010). NFAT deficient mice showed hypogenetic cardiac valve formation (de la Pompa et al. 1998; Ranger et al. 1998), while RCAN1.4 is not only a downstream target of calcineurin-NFAT signaling but also implicated in the regulation of calcineurin-NFAT activity (Wu et al. 2007). It suggests that RCAN1.4 probably play a complex role in the development of valve dysplasia. However, the exact role of RCAN1 in valve dysplasia needs to be further investigated.

## Challenges on drug development by targeting RCAN1

Considering its crucial roles in the pathogenesis of CVDs, RCAN1 may be a potential therapeutic target for CVDs treatment. However, there is no specific drug targeting RCAN1 developed in clinical trials, which may be attributed to the following reasons. First of all, RCAN1 has bidirectional roles in the regulation of calcineurin-NFAT signaling as mentioned above. Moreover, RCAN1 differentially regulated NFAT signaling in various microenvironments, such as microenvironment with different levels of AngII, PE, ET-1, PDGF or ISO (Shin et al. 2011). In addition, it is feasible and would be more precise to targeting specific isoform of RCAN1 for CVDs treatment as RCAN1.1 and RCAN1.4 have different N-terminal amino acids. However, the complex regulation and functional similarity and specificity of RCAN1 isoforms in CVDs remain elusive as aforementioned. For example, RCAN1.1 and RCAN1.4 are differently regulated by oxLDL (Mendez-Barbero et al. 2013). The homologous sequence of 168 amino acids at C-terminus within different RCAN1 isoforms may exert analogous functions. RCAN1.1 potentially protects against I/R injury by modulating mitochondrial function, while the functional role of RCAN1.4 in this process remains unclear (Parra et al. 2018).

The potential side effects of RCAN1 modulation should also be considered. Calcineurin inhibitors (CNIs) resembling RCAN1′s role in inhibiting calcineurin-NFAT signaling have been clinically applied as immunosuppressive agents, such as Cyclosporine A (CyA) and Tacrolimus (FK506) (Sharif et al. 2011; Xia et al. 2018). However, these CNIs could exert severe side effects such as hypertension (Robert et al. 2010), nephrotoxicity (Stempfle et al. 2002), hyperlipidemia (Taylor et al. 1999) and new-onset diabetes (Woodward et al. 2003). Meanwhile, RCAN1 plays both beneficial role and detrimental role in the pathogenesis of various diseases, such as various CVDs, cancer (Fu and Wu 2018), and Alzheimer’s disease (Wu et al. 2014). For instance, RCAN1.1 enhancement promotes stress-induced cell apoptosis (Sun et al. 2011; Wu and Song 2013). RCAN1.1 overexpression induces neuronal loss in hippocampus resulting in learning and memory deficits (Martin et al. 2012). Reduction of endogenous RCAN1 exacerbates cancer cell migration (Espinosa et al. 2009) and tumor growth (Wang et al. 2017). Therefore, both short-term and long-term side effects of targeting RCAN1 need to be considered and investigated.

## Conclusions

Mounting evidence has shown that RCAN1 plays crucial roles in the CVDs. Firstly, RCAN1 has a protective effect on myocardial I/R injury, myocardial hypertrophy and IMH/aortic rupture by modulating mitochondrial function, calcineurin activity and ROCK activity, respectively. Moreover, RCAN1 reduction protects against atherosclerosis by reducing oxLDL uptake. Furthermore, the role of RCAN1 in valve dysplasia needs to be further investigated. Although modulating RCAN1 expression may have therapeutic potential for CVDs, many challenges should be considered.

## Abbreviations

RCAN1: Regulator of calcineurin 1
CVDs: Cardiovascular diseases
NFAT: Nuclear factor of activated T cells
C/EBPβ: CCAAT/enhancer binding protein
TEF3: Transcription enhancer factor 3
AngII: Angiotensin II
PDGF: Platelet-derived growth factor
ISO: Isoproterenol
VEGF: Vascular endothelial growth factor
VEGFR2: Vascular endothelial growth factor receptor 2
LDLs: Low-density lipoproteins
PLCγ: Phospholipase C-γ
PIP2: Phosphatidylinositol (4,5)-bisphosphate
IP3: Inositol (1,4,5)-trisphosphate
DAG: Diacylglycerol
FHL2: Four-and-a-half LIM domain family protein 2
CyA: Cyclosporine A
AMI: Acute myocardial infarction
IMH: Intramural hematoma

## References

[CR1] Alghanem AF, Wilkinson EL, Emmett MS, Aljasir MA, Holmes K, Rothermel BA, Simms VA, Heath VL, Cross MJ (2017). RCAN1.4 regulates VEGFR-2 internalisation, cell polarity and migration in human microvascular endothelial cells. Angiogenesis.

[CR2] Baron T, Hambraeus K, Sundstrm J, Erlinge D, Jernberg T, Lindahl B, Grp T-AS (2016). Impact on long-term mortality of presence of obstructive coronary artery disease and classification of myocardial infarction. Am J Med.

[CR3] Burchfield JS, Xie M, Hill JA (2013). Pathological ventricular remodeling: mechanisms: part 1 of 2. Circulation.

[CR4] Carmeliet P, Ng YS, Nuyens D, Theilmeier G, Brusselmans K, Cornelissen I, Ehler E, Kakkar VV, Stalmans I, Mattot V (1999). Impaired myocardial angiogenesis and ischemic cardiomyopathy in mice lacking the vascular endothelial growth factor isoforms VEGF164 and VEGF188. Nat Med.

[CR5] Cereghetti GM, Stangherlin A, Martins de Brito O, Chang CR, Blackstone C, Bernardi P, Scorrano L (2008). Dephosphorylation by calcineurin regulates translocation of Drp1 to mitochondria. Proc Natl Acad Sci USA.

[CR6] Combs MD, Yutzey KE (2009). Heart valve development: regulatory networks in development and disease. Circ Res.

[CR7] Corbalan JJ, Kitsis RN (2018). RCAN1-calcineurin axis and the set-point for myocardial damage during ischemia-reperfusion. Circ Res.

[CR8] Cui PF, Liu X, Zhao K, Hou SQ, Chen C, Zhao DZ, Zeng HY (2020). The novel axis of YAP1, transcription enhancer factor 3 and Down Syndrome Candidate Region 1 isoform 1L is a common signaling pathway downstream of several angiogenic factors. Microvasc Res.

[CR9] de Asua DR, Quero M, Moldenhauer F, Suarez C (2015). Clinical profile and main comorbidities of Spanish adults with Down syndrome. Eur J Intern Med.

[CR10] de la Pompa JL, Timmerman LA, Takimoto H, Yoshida H, Elia AJ, Samper E, Potter J, Wakeham A, Marengere L, Langille BL (1998). Role of the NF-ATc transcription factor in morphogenesis of cardiac valves and septum. Nature.

[CR11] De Windt LJ, Lim HW, Bueno OF, Liang Q, Delling U, Braz JC, Glascock BJ, Kimball TF, del Monte F, Hajjar RJ (2001). Targeted inhibition of calcineurin attenuates cardiac hypertrophy in vivo. Proc Natl Acad Sci USA.

[CR12] Duan H, Li Y, Yan L, Yang H, Wu J, Qian P, Li B, Wang S (2015). Rcan1-1L overexpression induces mitochondrial autophagy and improves cell survival in angiotensin II-exposed cardiomyocytes. Exp Cell Res.

[CR13] Echtermeyer F, Harendza T, Hubrich S, Lorenz A, Herzog C, Mueller M, Schmitz M, Grund A, Larmann J, Stypmann J (2011). Syndecan-4 signalling inhibits apoptosis and controls NFAT activity during myocardial damage and remodelling. Cardiovasc Res.

[CR14] Elaimy AL, Mercurio AM (2018). Convergence of VEGF and YAP/TAZ signaling: Implications for angiogenesis and cancer biology. Sci Signal..

[CR15] Endemann G, Stanton LW, Madden KS, Bryant CM, White RT, Protter AA (1993). CD36 is a receptor for oxidized low density lipoprotein. J Biol Chem.

[CR16] Ermak G, Morgan TE, Davies KJ (2001). Chronic overexpression of the calcineurin inhibitory gene DSCR1 (Adapt78) is associated with Alzheimer's disease. J Biol Chem.

[CR17] Espinosa AV, Shinohara M, Porchia LM, Chung YJ, McCarty S, Saji M, Ringel MD (2009). Regulator of calcineurin 1 modulates cancer cell migration in vitro. Clin Exp Metastas.

[CR18] Esteban V, Mendez-Barbero N, Jimenez-Borreguero LJ, Roque M, Novensa L, Garcia-Redondo AB, Salaices M, Vila L, Arbones ML, Campanero MR (2011). Regulator of calcineurin 1 mediates pathological vascular wall remodeling. J Exp Med.

[CR19] Evangelista A, Dominguez R, Sebastia C, Salas A, Permanyer-Miralda G, Avegliano G, Elorz C, Gonzalez-Alujas T, Garcia Del Castillo H, Soler-Soler J (2003). Long-term follow-up of aortic intramural hematoma: predictors of outcome. Circulation.

[CR20] Fu Q, Wu Y (2018). RCAN1 in the inverse association between Alzheimer's disease and cancer. Oncotarget.

[CR21] Fuentes JJ, Pritchard MA, Planas AM, Bosch A, Ferrer I, Estivill X (1995). A new human gene from the Down syndrome critical region encodes a proline-rich protein highly expressed in fetal brain and heart. Hum Mol Genet.

[CR22] Fuentes JJ, Pritchard MA, Estivill X (1997). Genomic organization, alternative splicing, and expression patterns of the DSCR1 (Down syndrome candidate region 1) gene. Genomics.

[CR23] Garcia-Redondo AB, Esteban V, Briones AM, del Campo LSD, Gonzalez-Amor M, Mendez-Barbero N, Campanero MR, Redondo JM, Salaices M (2018). Regulator of calcineurin 1 modulates vascular contractility and stiffness through the upregulation of COX-2-derived prostanoids. Pharmacol Res.

[CR24] Guo RY, Li XF, Bai S, Guo J, Ding N, Li ZZ (2015). Association between DSCR1 variations and congenital heart disease susceptibility. Med Sci Monit.

[CR25] Guo L, Akahori H, Harari E, Smith SL, Polavarapu R, Karmali V, Otsuka F, Gannon RL, Braumann RE, Dickinson MH (2018). CD163+ macrophages promote angiogenesis and vascular permeability accompanied by inflammation in atherosclerosis. J Clin Invest.

[CR26] Han KA, Kang HS, Lee JW, Yoo L, Im E, Hong A, Lee YJ, Shin WH, Chung KC (2014). Histone deacetylase 3 promotes RCAN1 stability and nuclear translocation. PLoS ONE.

[CR27] Hansson GK, Hermansson A (2011). The immune system in atherosclerosis. Nat Immunol.

[CR28] Hashmi S, Al-Salam S (2015). Acute myocardial infarction and myocardial ischemia-reperfusion injury: a comparison. Int J Clin Exp Pathol.

[CR29] Hoffman JI, Kaplan S (2002). The incidence of congenital heart disease. J Am Coll Cardiol.

[CR30] Hojayev B, Rothermel BA, Gillette TG, Hill JA (2012). FHL2 binds calcineurin and represses pathological cardiac growth. Mol Cell Biol.

[CR31] Holmes K, Roberts OL, Thomas AM, Cross MJ (2007). Vascular endothelial growth factor receptor-2: structure, function, intracellular signalling and therapeutic inhibition. Cell Signal.

[CR32] Hunter JJ, Chien KR (1999). Signaling pathways for cardiac hypertrophy and failure. N Engl J Med.

[CR33] Iizuka M, Abe M, Shiiba K, Sasaki I, Sato Y (2004). Down syndrome candidate region 1, a downstream target of VEGF, participates in endothelial cell migration and angiogenesis. J Vasc Res.

[CR34] Jang GH, Park IS, Yang JH, Bischoff J, Lee YM (2010). Differential functions of genes regulated by VEGF-NFATc1 signaling pathway in the migration of pulmonary valve endothelial cells. FEBS Lett.

[CR35] Kaneko-Kawano T, Takasu F, Naoki H, Sakumura Y, Ishii S, Ueba T, Eiyama A, Okada A, Kawano Y, Suzuki K (2012). Dynamic regulation of myosin light chain phosphorylation by Rho-kinase. PLoS ONE.

[CR36] Kreusser MM, Lehmann LH, Keranov S, Hoting MO, Oehl U, Kohlhaas M, Reil JC, Neumann K, Schneider MD, Hill JA (2014). Cardiac CaM Kinase II genes delta and gamma contribute to adverse remodeling but redundantly inhibit calcineurin-induced myocardial hypertrophy. Circulation.

[CR37] Li X, Wang G, An Y, Li H, Li Y, Wu C (2015). Association between sequence variations in RCAN1 promoter and the risk of sporadic congenital heart disease in a Chinese population. Pediatr Cardiol.

[CR38] Li X, Shi L, Xu M, Zheng X, Yu Y, Jin J (2018). RCAN1 mutation and functional characterization in children with sporadic congenital heart disease. Pediatr Cardiol.

[CR39] Liu X, Zhao D, Qin L, Li J, Zeng H (2008). Transcription enhancer factor 3 (TEF3) mediates the expression of Down syndrome candidate region 1 isoform 1 (DSCR1-1L) in endothelial cells. J Biol Chem.

[CR40] Liu X, Zhao D, James L, Li J, Zeng H (2011). Requirement of the nuclear localization of transcription enhancer factor 3 for proliferation, migration, tube formation, and angiogenesis induced by vascular endothelial growth factor. FASEB J.

[CR41] Lusis AJ (2000). Atherosclerosis. Nature.

[CR42] Martin KR, Corlett A, Dubach D, Mustafa T, Coleman HA, Parkington HC, Merson TD, Bourne JA, Porta S, Arbones ML (2012). Over-expression of RCAN1 causes Down syndrome-like hippocampal deficits that alter learning and memory. Hum Mol Genet.

[CR43] Mendez-Barbero N, Esteban V, Villahoz S, Escolano A, Urso K, Alfranca A, Rodriguez C, Sanchez SA, Osawa T, Andres V (2013). A major role for RCAN1 in atherosclerosis progression. EMBO Mol Med.

[CR44] Molkentin JD, Lu JR, Antos CL, Markham B, Richardson J, Robbins J, Grant SR, Olson EN (1998). A calcineurin-dependent transcriptional pathway for cardiac hypertrophy. Cell.

[CR45] Murdoch JC, Rao SS, Fletcher CD, Dunnigan MG (1977). Down's syndrome: an atheroma-free model?. Br Med J.

[CR46] Nakata A, Nakagawa Y, Nishida M, Nozaki S, Miyagawa J, Nakagawa T, Tamura R, Matsumoto K, Kameda-Takemura K, Yamashita S (1999). CD36, a novel receptor for oxidized low-density lipoproteins, is highly expressed on lipid-laden macrophages in human atherosclerotic aorta. Arterioscl Throm Vas.

[CR47] Noh EH, Hwang HS, Hwang HS, Min B, Im E, Chung KC (2012). Covalent NEDD8 conjugation increases RCAN1 protein stability and potentiates its inhibitory action on calcineurin. PLoS ONE.

[CR48] Ong SB, Subrayan S, Lim SY, Yellon DM, Davidson SM, Hausenloy DJ (2010). Inhibiting mitochondrial fission protects the heart against ischemia/reperfusion injury. Circulation.

[CR49] Organization W.H. Global action plan for the prevention and control of NCDs 2013–2020. 2013. p. 55.

[CR50] Parra V, Altamirano F, Hernandez-Fuentes CP, Tong D, Kyrychenko V, Rotter D, Pedrozo Z, Hill JA, Eisner V, Lavandero S (2018). Down syndrome critical region 1 gene, Rcan1, helps maintain a more fused mitochondrial network. Circ Res.

[CR51] Peiris H, Raghupathi R, Jessup CF, Zanin MP, Mohanasundaram D, Mackenzie KD, Chataway T, Clarke JN, Brealey J, Coates PT (2012). Increased expression of the glucose-responsive gene, RCAN1, causes hypoinsulinemia, beta-cell dysfunction, and diabetes. Endocrinology.

[CR52] Pierpont ME, Basson CT, Benson DW, Gelb BD, Giglia TM, Goldmuntz E, McGee G, Sable CA, Srivastava D, Webb CL (2007). Genetic basis for congenital heart defects: current knowledge: a scientific statement from the American Heart Association Congenital Cardiac Defects Committee, Council on Cardiovascular Disease in the Young: endorsed by the American Academy of Pediatrics. Circulation.

[CR53] Qin L, Zhao D, Liu X, Nagy JA, Hoang MV, Brown LF, Dvorak HF, Zeng H (2006). Down syndrome candidate region 1 isoform 1 mediates angiogenesis through the calcineurin-NFAT pathway. Mol Cancer Res.

[CR54] Ranger AM, Grusby MJ, Hodge MR, Gravallese EM, de la Brousse FC, Hoey T, Mickanin C, Baldwin HS, Glimcher LH (1998). The transcription factor NF-ATc is essential for cardiac valve formation. Nature.

[CR55] Rateri DL, Davis FM, Balakrishnan A, Howatt DA, Moorleghen JJ, O'Connor WN, Charnigo R, Cassis LA, Daugherty A (2014). Angiotensin II induces region-specific medial disruption during evolution of ascending aortic aneurysms. Am J Pathol.

[CR56] Robert N, Wong GWK, Wright JM (2010). Effect of cyclosporine on blood pressure. Cochrane Database Syst Rev..

[CR57] Rodrigues A, Coelho L, Goncalves W, Vasconcellos M, Cunha R, Gouvea S, Abreu GJVH, Management R (2011). Stiffness of the large arteries in individuals with and without Down syndrome. Vasc Health Risk Manag.

[CR58] Rotter D, Grinsfelder DB, Parra V, Pedrozo Z, Singh S, Sachan N, Rothermel BA (2014). Calcineurin and its regulator, RCAN1, confer time-of-day changes in susceptibility of the heart to ischemia/reperfusion. J Mol Cell Cardiol.

[CR59] Roy-Vallejo E, Galvan-Roman JM, Moldenhauer F, de Asua DR (2020). Adults with Down syndrome challenge another paradigm: When aging no longer entails arterial hypertension. J Clin Hypertens.

[CR60] Sanna B, Brandt EB, Kaiser RA, Pfluger P, Witt SA, Kimball TR, van Rooij E, De Windt LJ, Rothenberg ME, Tschop MH (2006). Modulatory calcineurin-interacting proteins 1 and 2 function as calcineurin facilitators in vivo. Proc Natl Acad Sci USA.

[CR61] Scherz-Shouval R, Elazar Z (2011). Regulation of autophagy by ROS: physiology and pathology. Trends Biochem Sci.

[CR62] Sharif A, Shabir S, Chand S, Cockwell P, Ball S, Borrows R (2011). Meta-analysis of calcineurin-inhibitor-sparing regimens in kidney transplantation. J Am Soc Nephrol.

[CR63] Shen L, Oshida T, Miyauchi J, Yamada M, Miyashita T (2004). Identification of novel direct transcriptional targets of glucocorticoid receptor. Leukemia.

[CR64] Shin SY, Yang HW, Kim JR, Do Heo W, Cho KH (2011). A hidden incoherent switch regulates RCAN1 in the calcineurin-NFAT signaling network. J Cell Sci.

[CR65] Song HK, Hong SE, Kim T, Kim DH (2012). Deep RNA sequencing reveals novel cardiac transcriptomic signatures for physiological and pathological hypertrophy. PLoS ONE.

[CR66] Stempfle HU, Hien M, Meiser B, Frost R, Theisen K (2002). Tacrolimus (FK506) versus cyclosporine nephrotoxicity in heart transplant recipients: a four-year follow-up. J Am Coll Cardiol.

[CR67] Sun XL, Wu YL, Chen B, Zhang ZH, Zhou WH, Tong YG, Yuan JY, Xia K, Gronemeyer H, Flavell RA (2011). Regulator of calcineurin 1 (RCAN1) facilitates neuronal apoptosis through caspase-3 activation. J Biol Chem.

[CR68] Sun XL, Wu YL, Herculano B, Song WH (2014). RCAN1 overexpression exacerbates calcium overloading-induced neuronal apoptosis. PLoS ONE.

[CR69] Sun L, Hao Y, An R, Li H, Xi C, Shen G (2014). Overexpression of Rcan1-1L inhibits hypoxia-induced cell apoptosis through induction of mitophagy. Mol Cells.

[CR70] Sussman MA, Lim HW, Gude N, Taigen T, Olson EN, Robbins J, Colbert MC, Gualberto A, Wieczorek DF, Molkentin JD (1998). Prevention of cardiac hypertrophy in mice by calcineurin inhibition. Science.

[CR71] t Hoen PA, Van der Lans CA, Van Eck M, Bijsterbosch MK, Van Berkel TJ, Twisk J (2003). Aorta of ApoE-deficient mice responds to atherogenic stimuli by a prelesional increase and subsequent decrease in the expression of antioxidant enzymes. Circ Res.

[CR72] Takahashi T, Yamaguchi S, Chida K, Shibuya M (2001). A single autophosphorylation site on KDR/Flk-1 is essential for VEGF-A-dependent activation of PLC-gamma and DNA synthesis in vascular endothelial cells. EMBO J.

[CR73] Tan N, Chung MK, Smith JD, Hsu J, Serre D, Newton DW, Castel L, Soltesz E, Pettersson G, Gillinov AM (2013). Weighted gene coexpression network analysis of human left atrial tissue identifies gene modules associated with atrial fibrillation. Circ Cardiovasc Genet.

[CR74] Taylor DO, Barr ML, Radovancevic B, Renlund DG, Mentzer RM, Smart FW, Tolman DE, Frazier OH, Young JB, VanVeldhuisen P (1999). A randomized, multicenter comparison of tacrolimus and cyclosporine immunosuppressive regimens in cardiac transplantation: Decreased hyperlipidemia and hypertension with tacrolimus. J Heart Lung Transpl.

[CR75] Thomas H, Diamond J, Vieco A, Chaudhuri S, Shinnar E, Cromer S, Perel P, Mensah GA, Narula J, Johnson CO (2018). Global Atlas of Cardiovascular Disease 2000–2016: the path to prevention and control. Glob Heart.

[CR76] Uniewicz KA, Cross MJ, Fernig DG (2011). Exogenous recombinant dimeric neuropilin-1 is sufficient to drive angiogenesis. J Biol Chem.

[CR77] van Rooij E, Doevendans PA, Crijns HJGM, Heeneman S, Lips DJ, van Bilsen M, Williams RS, Olson EN, Bassel-Duby R, Rothermel BA (2004). MCIP1 overexpression suppresses left ventricular remodeling and sustains cardiac function after myocardial infarction. Circ Res.

[CR78] Vega RB, Rothermel BA, Weinheimer CJ, Kovacs A, Naseem RH, Bassel-Duby R, Williams RS, Olson EN (2003). Dual roles of modulatory calcineurin-interacting protein 1 in cardiac hypertrophy. Proc Natl Acad Sci USA.

[CR79] Viles-Gonzalez JF, Fuster V, Badimon JJ (2004). Atherothrombosis: A widespread disease with unpredictable and life-threatening consequences. Eur Heart J.

[CR80] Villahoz S, Yunes-Leites PS, Mendez-Barbero N, Urso K, Bonzon-Kulichenko E, Ortega S, Nistal JF, Vazquez J, Offermanns S, Redondo JM (2018). Conditional deletion of Rcan1 predisposes to hypertension-mediated intramural hematoma and subsequent aneurysm and aortic rupture. Nat Commun.

[CR81] Wang CJ, Saji M, Justiniano SE, Yusof AM, Zhang XL, Yu LB (2017). RCAN1-4 is a thyroid cancer growth and metastasis suppressor. Jci Insight..

[CR82] Woodward RS, Schnitzler MA, Baty J, Lowell JA, Lopez-Rocafort L, Haider S, Woodworth TG, Brennan DC (2003). Incidence and cost of new onset diabetes mellitus among U.S. wait-listed and transplanted renal allograft recipients. Am J Transplant.

[CR83] Wu YL, Song WH (2013). Regulation of RCAN1 translation and its role in oxidative stress-induced apoptosis. FASEB J.

[CR84] Wu H, Kao SC, Barrientos T, Baldwin SH, Olson EN, Crabtree GR, Zhou B, Chang CP (2007). Down syndrome critical region-1 is a transcriptional target of nuclear factor of activated T cells-c1 within the endocardium during heart development. J Biol Chem.

[CR85] Wu Y, Ly PT, Song W (2014). Aberrant expression of RCAN1 in Alzheimer's pathogenesis: a new molecular mechanism and a novel drug target. Mol Neurobiol.

[CR86] Xia TY, Zhu S, Wen Y, Gao SH, Li MM, Tao X, Zhang F, Chen WS (2018). Risk factors for calcineurin inhibitor nephrotoxicity after renal transplantation: a systematic review and meta-analysis. Drug Des Dev Ther.

[CR87] Xie M, Burchfield JS, Hill JA (2013). Pathological ventricular remodeling: therapies: part 2 of 2. Circulation.

[CR88] Yan L, Yang H, Li Y, Duan H, Wu J, Qian P, Li B, Wang S (2014). Regulator of calcineurin 1–1L protects cardiomyocytes against hypoxia-induced apoptosis via mitophagy. J Cardiovasc Pharmacol.

[CR89] Yan D, He Y, Dai J, Yang L, Wang X, Ruan Q (2017). Vascular endothelial growth factor modified macrophages transdifferentiate into endothelial-like cells and decrease foam cell formation. Biosci Rep..

[CR90] Yang J, Rothermel B, Vega RB, Frey N, McKinsey TA, Olson EN, Bassel-Duby R, Williams RS (2000). Independent signals control expression of the calcineurin inhibitory proteins MCIP1 and MCIP2 in striated muscles. Circ Res.

[CR91] Yang H, Zhou L, Wang Z, Roberts LJ, Lin X, Zhao Y, Guo Z (2009). Overexpression of antioxidant enzymes in ApoE-deficient mice suppresses benzo(a)pyrene-accelerated atherosclerosis. Atherosclerosis.

[CR92] Ylä-Herttuala S, Nikkari T, Kivimäki T (1989). Down's syndrome and atherosclerosis. Atherosclerosis.

[CR93] Yoshida NL, Miyashita T, Yamada M, Reed JC, Sugita Y, Oshida T (2002). Analysis of gene expression patterns during glucocorticoid-induced apoptosis using oligonucleotide arrays. Biochem Biophys Res Commun.

[CR94] Zou Y, Hiroi Y, Uozumi H, Takimoto E, Toko H, Zhu W, Kudoh S, Mizukami M, Shimoyama M, Shibasaki F (2001). Calcineurin plays a critical role in the development of pressure overload-induced cardiac hypertrophy. Circulation.

